# Misdiagnosis of COVID-19 infection before molecular confirmation in Sulaimaniyah City, Iraq

**DOI:** 10.1186/s40001-022-00704-0

**Published:** 2022-06-03

**Authors:** Hemn Muhammed Mustafa, Darya Saeed Abdulateef, Heshu Sulaiman Rahman

**Affiliations:** 1 Department of Medicine, College of Medicine, University of Sulaimani, Sulaimani New, Street 29, Zone 207, Sulaymaniyah, 46001 Republic of Iraq; 2 Department of Physiology, College of Medicine, University of Sulaimani, Sulaimani New, Street 29, Zone 207, Sulaymaniyah, 46001 Republic of Iraq; 3 Department of Medical Laboratory Sciences, Komar University of Science and Technology, Sulaimaniyah, Iraq

**Keywords:** COVID-19, Misdiagnosis, Molecular diagnosis, Non-physician healthcare workers, Iraq

## Abstract

**Background:**

During the last 2 years, in the Kurdistan Region, Northern Iraq, there were thousands of COVID-19 cases that have not been reported officially, but diagnosed and confirmed by private laboratories and private hospitals, or clinicians based on typical clinical signs, as well as few people using home self-test after appearing of some flu-like clinical symptoms. Thus, this study aims to assess the misdiagnosis and mismanagement of cases before COVID-19 confirmation.

**Methods:**

This study enrolled 100 consecutive patients who visited an outpatient clinic of Shar Hospital that had symptoms highly suspicious of COVID-19 infection while misdiagnosed previously to have other types of disease. Detailed questionnaires were filled for all studied patients, including age, gender, main presenting symptoms, and duration of these symptoms with the following questions: who made the false diagnosis, depending on which diagnostic test the false diagnosis was made, which medication was used for the false diagnosis, who prescribed those medications, and how long those medications were used. They were investigated by RT-PCR on their nasopharyngeal swab for confirmation.

**Results:**

Most of the false diagnoses were typhoid (63%), influenza (14%), pneumonia (9%), gastroenteritis (5%), common cold (4%), brucellosis (4%), and meningitis (1%). Regarding the false diagnosis of cases, 92% were made by non-physician healthcare workers, and only 8% were made by physicians. All false diagnoses with typhoid, gastroenteritis, and common cold were made by non-physician healthcare workers, together with about half of the diagnosis of pneumonia and brucellosis, with statistically significant results (*P* < 0.001).

**Conclusions:**

We realized that some patients had been misdiagnosed before the COVID-19 infection confirmation. Their health conditions improved drastically after correct diagnosis and treatment, and this research is considered the first research to be conducted in Iraq in this regard.

## Introduction

COVID-19 is an infectious pandemic disease caused by severe acute respiratory syndrome coronavirus-2 (SARS-CoV-2) [1]. Up to date, scientists are trying to identify a new antiviral specific drug to overcome this disease. Different methods are under study and evaluation worldwide to control the virus, including blood plasma, blood purification, antimicrobial, and antiviral agents; though, there are no approved drugs yet [2, 3].

The COVID-19 virus emerged in December 2019 in Wuhan city, Hubei Province, China, and then spread rapidly worldwide. An ongoing outbreak of pneumonia associated with emerging viral infections to global public health is called SARS-CoV-2 [4, 5].

Up to now, six species of coronavirus are well known to cause human diseases, whereas four species lead to diseases in humans and causing common cold in immunocompetent individuals, including alpha-Coronaviruses (HCoV-229E and HCoV-NL63) and beta-Coronaviruses (HCoV-HKU1 and HCoV-OC43) [6]. While the other two species are zoonotic infectious agents named the Middle East Respiratory Syndrome coronavirus (MERS-CoV) and severe acute respiratory syndrome coronavirus (SARS-CoV) [7]. Back in 2003 and 2012, the causative agent of the SARS-CoV was isolated from an outbreak in China, while MERS-CoV was from an outbreak in Saudi Arabia in 2012 [8].

Despite some diversity in initial symptoms, generally, the reported clinical features of confirmed COVID-19 patients are fever and respiratory symptoms such as cough, dyspnea, shortness of breath, sore throat, and sputum formation, as well as myalgia, headache, hemoptyses, and gastrointestinal symptoms, like nausea, abdominal pain, vomiting, and diarrhea have been seen [9, 10]. Up to date, it is not understood why some patients infected with SARS-CoV-2 have mild or even asymptomatic diseases, but others develop severe by the same infectious virus [11].

Some hospitalized patients showed bilateral lung ground-glass opacity on computed tomography (CT) imaging. In contrast, particular patients in the intensive care unit (ICU) progressed to acute respiratory distress syndrome (ARDS) or acute kidney injury. Few of them developed a secondary bacterial infection (pneumonia), shock, and might result in death [12, 13]. Most of the patients in ICU required ventilator and oxygen therapy, anti-inflammatory, antibacterial, and antiviral therapies, or even plasma transferring from recovered peoples [14].

In the Kurdistan region in Northern Iraq, the first case appeared on March 1, 2020, and up to date (September 25, 2021), 325,432 confirmed COVID-19 cases (291,882 recovered, 5795 deaths, and 27,755 active cases) had been reported by the Ministry of Health/Kurdistan Regional Government (KRG). Most cases are asymptomatic carriers; some show mild-to-moderate clinical signs, while a minority have severe symptoms [15].

The number of confirmed cases in Kurdistan/Iraq is increasing rapidly; people with severe cases visited the public hospitals for diagnosis, hospitalization, and treatment, while those with moderate symptoms either visited the hospitals or non-physician healthcare workers to be diagnosed, treated, and recommended. However, asymptomatic carriers and individuals with minor symptoms either quarantined themselves at home or avoided visiting public areas to reduce others’ exposure to the disease. Moreover, those who visited non-physician healthcare workers were mainly misdiagnosed and mistreated for other diseases such as typhoid fever, brucellosis, pneumonia, influenza, common cold, gastroenteritis, and meningitis. Thus, in this research, we addressed to assess the false positivity of typhoid serology in COVID-19 confirmed cases to avoid misdiagnosis and mistreatment and consequently improve the health conditions of COVID-19 patients.

## Methods and materials

### Inclusion criteria

Adult patients who were highly suspicious of COVID-19, whose RT-PCR test was positive using a nasopharyngeal swab, previously diagnosed falsely with other infectious diseases rather than COVID-19, and received mistreatment for the misdiagnosed disease, were included.

### Exclusion criteria

The patients with negative RT-PCR tests or suspected to have a disease other than COVID-19 infection were excluded.

### Patients and methodology

This study enrolled 100 consecutive patients (60 males and 40 females) who visited an outpatient clinic of Shar Hospital. They had symptoms highly suspicious of COVID-19 infection while misdiagnosed previously to have other types of disease.

Detailed questionnaires were filled for all studied patients, including a clear history (age, gender, main presenting symptoms, and duration of these symptoms). Additionally, the following questions were asked from all the patients: who made the false diagnosis, depending on which diagnostic test the false diagnosis was made, which medication was used for the false diagnosis, who prescribed those medications, and how long those medications were used. All the answers to the above questions were reported, and their questionnaires were coded. All patients gave a history of false diagnosis before the presentation to the hospital and before confirming COVID-19 infection.

The laboratory records of those patients were checked, and they revealed that their diagnosis with typhoid fever was dependent on typhoid IgM/IgG rapid test, diagnosis of brucellosis was based on Rose Bengal test, chest X-ray finding was used for the pneumonia diagnosis, while the diagnosis of gastroenteritis, influenza and common cold were only based on clinical presentations.

All who had a history and symptoms highly suspicious for COVID-19 infection were investigated by RT-PCR on their nasopharyngeal swab for confirmation, and the patients with positive results for COVID-19 were included in this study. Finally, all patients were evaluated thoroughly, and the decision of hospitalization or home management was made by an expert physician.

### Statistical analysis

All data were entered into SPSS software version 22, and coding of the variables was done. The descriptive parameters, age, and duration of treatment used till confirmation of COVID-19 were shown as mean ± SD. All remaining data were categorical variables and evaluated as frequency and percentages. The patients were grouped into seven groups according to the false diagnosis, and the comparisons in descriptive parameters between these groups were performed using one-way ANOVA and post hoc test. The Chi-square test was used to find association and comparison between categorical variables. The significant statistical value was set at 0.05. A stacked bar chart was used to demonstrate the frequency (percentage) of each symptom in the studied participants. The box plot was used to compare the mean age between hospitalized and non-hospitalized patients and also between dead and alive patients. Mann–Whitney *T*-test was used to find significant statistical value, and *P* < 0.05 was regarded as significant.

## Results

In this study, 100 subjects who were confirmed to have COVID-19 based on RT-PCR of nasopharyngeal swabs were falsely diagnosed previously. Most of the false diagnoses were typhoid fever (63%), followed by influenza (14%) and pneumonia (9%). Few of them were also falsely diagnosed as having gastroenteritis (5%), common cold (4%), brucellosis (4%), and meningitis (1%) (Table 1). The mean age of the participants of COVID-19 confirmed patients with the previous false diagnosis was 42.34 ± 15.56 years, and most of the patients were male (60%) (Table 1).

**Table 1:** General and clinical characteristics of the studied participants according to types of misdiagnosis before COVID-19 confirmations

	Types of misdiagnosis^a^							*P*-value
	Total	Typhoid	Influenza	Pneumonia	Gastro-enteritis	Common cold	Brucellosis	
Total no.	100	63	14	9	5	4	4	
Parameters	N (%) mean (SD)	N (%) mean (SD)	N (%) mean (SD)	N (%) mean (SD)	N (%) mean (SD)	N (%) mean (SD)	N (%) mean (SD)	
Age^b^	42.34 (15.56)	41.21 (15.5)	44.36 (14.04)	52.56 (11.61)	38.4 (14.69)	43.25 (18.75)	29.25 (20.65)	0.159
Sex								
Male	60 (60)	37 (58.7)	7 (50)	7 (77.8)	2 (40)	2 (50)	4 (100)	0.407
Female	40 (40)	26 (41.3)	7 (50)	2 (22.2)	3 (60)	2 (50)	0 (0.0)	
Investigation based on								
Typhoid IgM and IgG rapid test	63 (63)	63 (100)	0 (0)	0 (0)	0 (0)	0 (0)	0 (0)	< 0.001
Rose Bengal	4(4)	0 (0)	0 (0)	0 (0)	0 (0)	0 (0)	4 (100)	
Chest X-ray	5 (5)	0 (0)	0 (0)	5 (55.6)	0 (0)	0 (0)	0 (0)	
None (clinical presentation)	28 (28)	0 (0)	14 (100)	4 (44.4)	5 (100)	4 (100)	0 (0)	
MisDX made by								
Non-physician healthcare worker	92 (92)	63 (100)	14 (100)	4 (44.4)	5 (100)	4 (100)	2 (50)	< 0.001
Physician	8 (8)	0 (0)	0 (0)	5 (55.6)	0 (0)	0 (0)	2 (50)	
Duration of the mistreatment (days)	5.44 (1.733)	5.63 (1.47)	4.21 (1.12)	6 (2.55)	4.4 (1.67)	5.75 (1.5)	7.25 (2.5)	0.002
Hospitalized	28 (28)	15 (23.8)	4 (28.6)	2 (22.2)	2 (40)	3 (75)	1 (25)	0.236
Died^a^	4 (4)	1 (1.6)	2 (14.3)	1 (11.1)	0 (0)	0 (0)	0 (0)	0.361

^a^One patient was diagnosed with meningitis by a physician, its data is not shown in this table

^b^Descriptive parameters were shown as mean (SD). All other parameters were categorical variable shown as numbers (percentages)

All the patients who were falsely diagnosed with typhoid, their diagnoses were made based on positive typhoid IgM and IgG rapid tests, and who were falsely diagnosed with brucellosis based on positive Rose Bengal tests. All falsely diagnosed pneumonia was based on a chest X-ray. In contrast, all other patients who were falsely diagnosed to have influenza, common cold, and gastroenteritis, the diagnosis was made based on clinical presentations. All these results were statistically significant (Table 1).

Most (92%) of the false diagnoses were made by non-physician healthcare workers, and only 8% of the false diagnosis were made by physicians. All false diagnoses with typhoid fever, gastroenteritis, and common cold were made by non-physician healthcare workers, together with about half of the diagnosis of pneumonia and brucellosis, with statistically significant results (*P* < 0.001). One patient, 62-year-old male, was falsely diagnosed by a physician as having meningitis because fever, headache, and confusion were the main presenting symptoms; later, after 2 days, he was confirmed with RT-PCR for having COVID-19. The mean duration of receiving treatment for the false diagnosis till the COVID-19 diagnosis was 5.44 days (Table 1).

Among the studied patients with false diagnoses, the hospitalization rate was 28%, and the rate of death was 4%. There are no significant differences in the rate of death and hospitalization in patients who were diagnosed falsely with different diseases before confirmation with COVID-19 (Table 1).

**Fig. 1:**
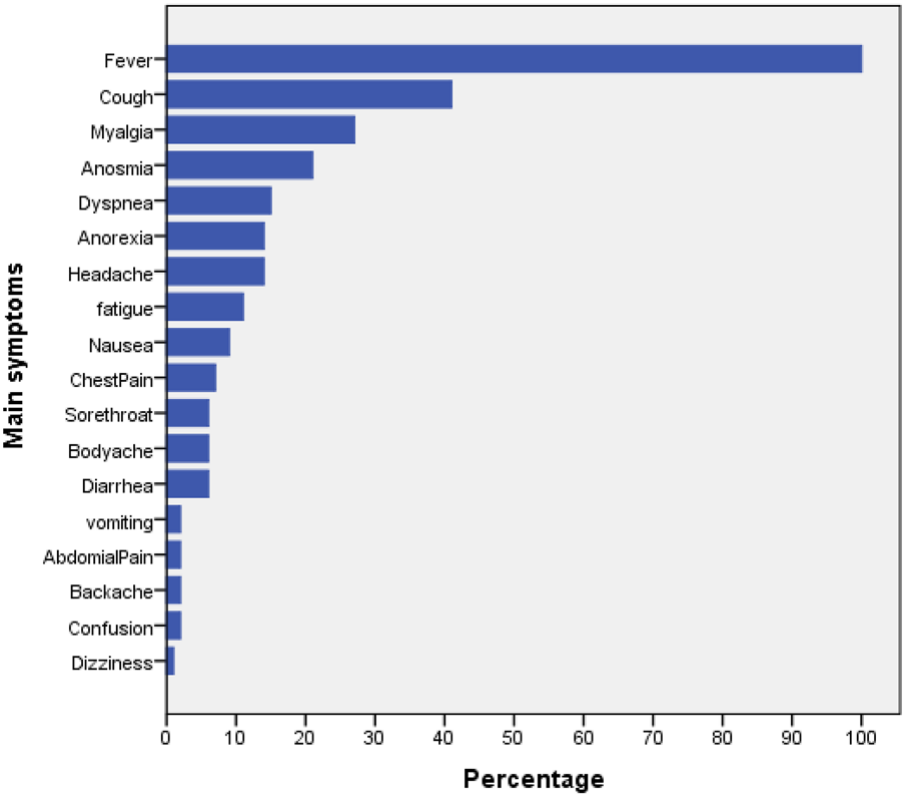
Percentages of the main presenting symptoms in confirmed COVID-19 infected patients

The most frequent symptoms present in all patients were fever (100%), followed by cough (41%), myalgia (27%), and anosmia (21%) (Fig. 1). The most frequent medication used to treat patients with a false diagnosis before COVID-19 confirmation is revealed in Table 2. The ceftriaxone vial was the most frequently prescribed medication to the false diagnosed typhoid patients (65%).

**Table 2:** The most frequent medication (mistreatment) used to treat the patients based on the misdiagnosis

False diagnosis	Typhoid	Influenza	Pneumonia	Gastro-enteritis	Common cold	Brucellosis
Number	63	14	9	5	4	4
Most frequent medication	Ceftriaxone vial	Dexamethasone combined with diclofenac sodium	Levofloxacin Tab	Metronidazole	Amoxiclav	Rifampicin with doxycycline
No. (%)	41 (65%)	9 (64.3)	3 (33.3%)	5 (100%)	3 (75%)	4 (100%)

**Fig. 2:**
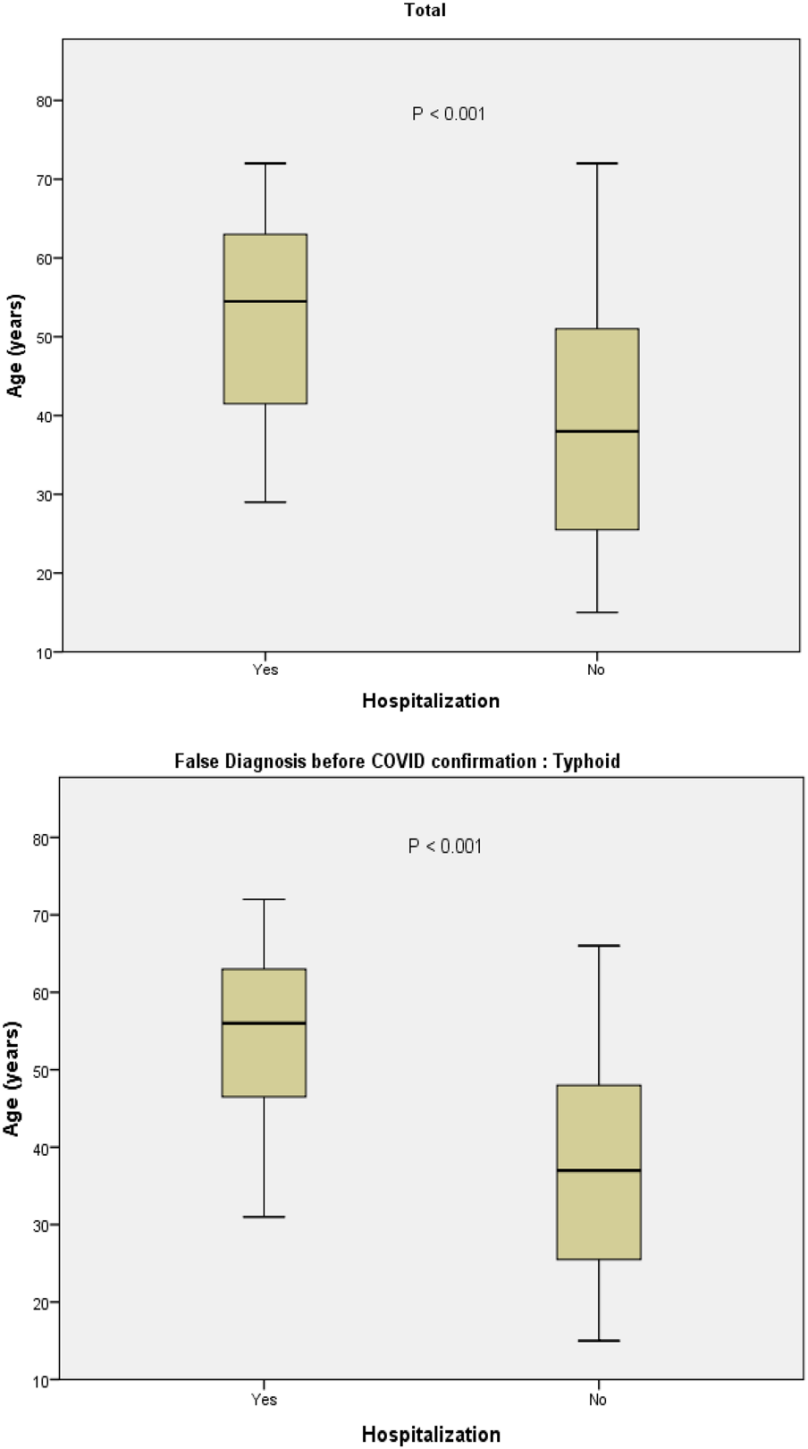
Mean age compared between hospitalized and non-hospitalized patients, in the **a** total confirmed COVID-19 infected patients with previous misdiagnosis and, **b** COVID-19 infected patients with false typhoid fever diagnosis

Figure 2 shows a comparison between mean age in patients who were hospitalized and non-hospitalized patients. The mean age of patients who were hospitalized was significantly higher compared to non-hospitalized patients. The same was true in COVID-19 patients with false diagnosed typhoid (*P* < 0.001).

**Fig. 3:**
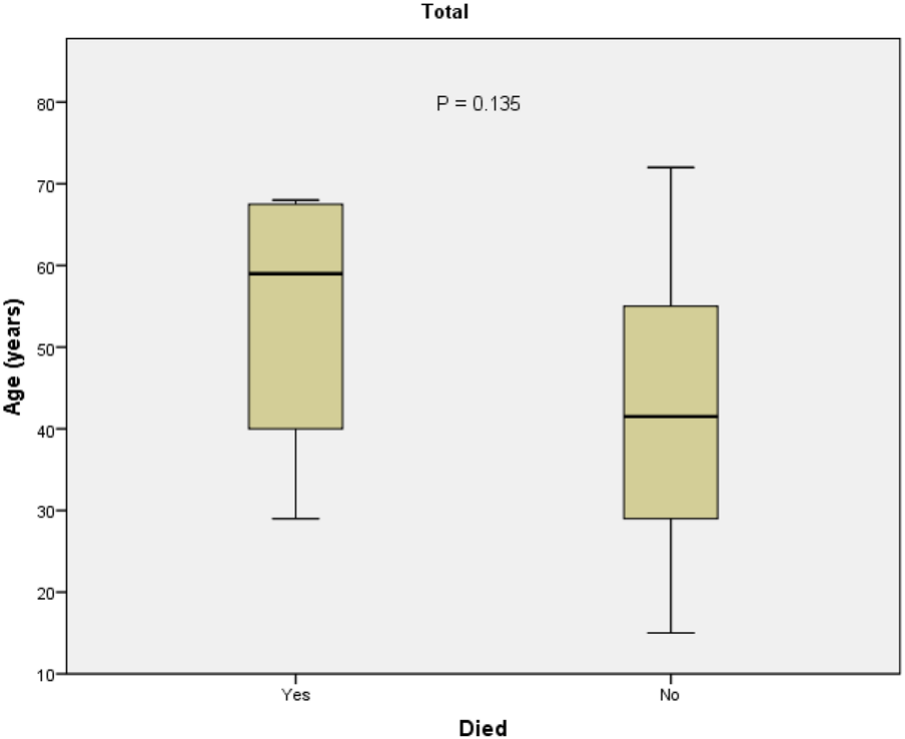
Mean age compared between dead and live patients, in the misdiagnosed COVID-19 confirmed patients

As shown in Fig. 3, the mean age of patients who died was higher than the mean age of patients alive from the disease, but the data failed to reach a significant level statistically. The delay in the diagnosis of COVID-19 does not show a significant correlation with the rate of hospitalization and death (*P* < 0.05).

## Discussion

In the Kurdistan region, Northern Iraq, after appearing of the first COVID-19 case, every effort by health teams and government-run security agencies promptly and seriously was intensified to quarantine citizens in their homes, blocked traffic in all directions, banned travels between the Kurdistan cities and between the Kurdistan region and federal Iraqi provinces for about 60 days to prevent the virus’s spread [11]. This was followed by a silent period with very few or even no reported cases for about 2 weeks which made the people force the KRG to lift the lockdown and curfew. After that, the COVID-19 infection has started again, and hundreds (some days reached more than a thousand) of cases were reported daily that was associated with higher severity of clinical signs, increased hospitalization rate, and also higher mortality rate, with various treatment choices, and outcomes of the disease in comparison to the previous cases. The majority of cases are asymptomatic carriers, and some show mild-to-moderate clinical signs, while the minority has severe debilitating symptoms [16].

Despite all these facilities and services offered to COVID-19 patients in Public Hospitals in various areas of the Kurdistan region, there are still some misdiagnosed and mistreated COVID-19 cases, especially by non-physician healthcare workers, that lead to sad consequences.

In the Kurdistan region or even Iraq, there is no published data about the misdiagnosis and mistreatment information of the COVID-19 patients with other infectious diseases, especially typhoid fever, influenza, pneumonia, gastroenteritis, common cold, brucellosis, and meningitis.

In this current study, we realized that men are misdiagnosed more often than women which might be due to men being more commonly infected with COVID-19 than women [17], since the majority of outdoor works are done by men (business, running shops, employee, and office works). Additionally, we emphasized that the majority of misdiagnosed people are aged above 40 years, which might be due to the fact the disease is more common in adult mature individuals or the condition is mostly affected peoples with comorbidities such as hypertension, diabetes, cancer, hypocholesteremia which are more common in older people than younger ones [18].

On the other hand, among the infections misdiagnosed with COVID-19 cases, typhoid fever is the commonest, while meningitis is the less common one. This result might be due to that the typhoid fever is endemic in our region, and annually too many peoples being diagnosed with this disease in all age groups for both sexes [19]. Another factor is that the typhoid serology is not as sensitive as detecting the specific typhoid IgM and IgG antibodies and globally; this diagnostic test is no more recommended for typhoid fever diagnosis [20].

In our region, because of a severe economic crisis there are too many non-physician healthcare workers who have established a local clinic in neighborhoods for diagnostic evaluations, therapeutic remediation, surgical assistance, and pharmaceutical prescription without having sufficient skills in those fields, and this mostly results in misleading sick people that results in misdiagnosis and mistreatment of patients. Regarding the patients with COVID-19 infections, in a total of 100 positive cases, 92 cases were misdiagnosed and treated by non-physician healthcare workers in which some patients’ life ended with death.

About the clinical signs of COVID-19 misdiagnosed patients, the majority of diseases people presented fever which might be due to that the fever is a common clinical sign of all infectious diseases including viral infections [21]. At the same time, dizziness was reported to be a less common symptom.

Furthermore, ceftriaxone is commonly used as a treatment of choice by misdiagnosed COVID-19 patients. At the same time, amoxiclav is a less commonly used antibiotic, which might be due to that ceftriaxone is a broad-spectrum antibiotic that is most commonly used for the treatment of typhoid fever [22]. In this study, most COVID-19 cases were misdiagnosed with typhoid fever.

## Conclusion

In conclusion, we realized that non-physician healthcare workers are the primary cause of misdiagnosis and mistreatment of COVID-19 patients in Sulaimaniyah city, Kurdistan Region, Iraq. Additionally, a single typhoid IgM and IgG rapid test are neither a specific nor a sensitive diagnostic option for the diagnosis of typhoid fever. It gives a high pseudo-positive result in people infected with other diseases and not typhoid fever, leading to misdiagnosis followed by mistreatment, especially in COVID-19 patients.

## Data Availability

All data generated or analyzed during this study are included in this published article.

## Abbreviations

ARDS: Acute respiratory distress syndrome
CT: Computed tomography
COVID-19: Corona virus disease-2019
ICU: Intensive care unit
IgM: Immunoglobulin M
KRG: Kurdistan Regional Government
RT-PCR: Real-time polymerase chain reaction
SARS: Severe acute respiratory syndrome coronavirus-2
WHO: World Health Organization
